# Randomized, phase I/II study of gemcitabine plus IGF-1R antagonist (MK-0646) versus gemcitabine plus erlotinib with and without MK-0646 for advanced pancreatic adenocarcinoma

**DOI:** 10.1186/s13045-018-0616-2

**Published:** 2018-05-30

**Authors:** Reham Abdel-Wahab, Gauri R. Varadhachary, Priya R. Bhosale, Xuemei Wang, David R. Fogelman, Rachna T. Shroff, Michael J. Overman, Robert A. Wolff, Milind Javle

**Affiliations:** 1 Department of Gastrointestinal Medical Oncology, The University of Texas MD Anderson Cancer Center, 1515 Holcombe Blvd, Unit 426, Houston, TX 77030 USA; 2 Department of Diagnostic Radiology, The University of Texas MD Anderson Cancer Center, Houston, TX USA; 3 Department of Biostatistics, The University of Texas MD Anderson Cancer Center, Houston, TX USA; 4 Clinical Oncology Department, Assiut University Hospitals, Assiut, Egypt

**Keywords:** MK-0646, Gemcitabine, Erlotinib, Advanced pancreatic adenocarcinoma

## Abstract

**Background:**

Binding of insulin-like growth factor-I (IGF-1) to its receptor (IGF-1R) initiates downstream signals that activate PI3K/Akt/mTOR and MEK/Erk pathways, which stimulate cancer cell proliferation and induce drug resistance. Cross talk between IGF-1R and epidermal growth factor receptor (EGFR) mediates resistance to anti-EGFR agents. We studied safety, tolerability, and outcomes of MK-0646, IGF-1 monoclonal antibody, in combination with gemcitabine (G) ± erlotinib (E) in metastatic pancreatic cancer.

**Methods:**

Our study included a phase I dose escalation and phase II randomization and expansion cohorts. A 3 + 3 dose escalation protocol was used to determine MK-0646 maximum tolerable dose (MTD) in combination with G ± E standard doses. For phase II, patients were randomized to arm A (G + MK), arm B (G + MK + E), or arm C (G + E). Primary endpoint was progression-free survival (PFS). Secondary endpoints were overall survival (OS), disease control rate, toxicity, and correlation between OS and IGF-1 in patients treated with MK-0646.

**Results:**

MK-0646 MTD was 10 mg/kg in combination with G and 5 mg/kg in combination with G + E. In randomization cohort, 15 patients were treated in each arm. Disease control rates were 50, 60, and 40% respectively. PFS was not different between the three arms. OS was significantly different between arm A (10.4 months) and C (5.7 months) (*P* = 0.02). However, addition of erlotinib in arm B yielded no OS benefit compared to arm A (*P* = 0.6). Plasma and tissue IGF-1 levels did not correlate with OS (*P* = 0.64, 0.87). Grade 3–4 toxicity during phase II cohorts were neutropenia (10/arm A, 14/arm B, 5/arm C), leukopenia (5/A, 5/B, 7/C), thrombocytopenia (8/A, 9/B, 2/C), hyponatremia (1/A, 3/B), and hyperglycemia (8/A, 1/B).

**Conclusions:**

MK-0646 was tolerable in combination with G and associated with improvement in OS but not PFS as compared with G + E. Tissue and serum IGF-1 did not correlate with clinical outcome.

**Trial registration:**

This trial is registered in ClinicalTrial.gov under the Identifier NCT00769483 and registration date was October 9, 2008.

**Electronic supplementary material:**

The online version of this article (10.1186/s13045-018-0616-2) contains supplementary material, which is available to authorized users.

## Background

Pancreatic cancer (PCA) is an aggressive disease with < 1-year median overall survival (OS). Gemcitabine provided a survival advantage over 5-fluorouracil for advanced disease stage [1]. Combination regimens including gemcitabine/nab-paclitaxel or 5-fluorouracil/leucovorin/irinotecan/oxaliplatin (FOLFIRINOX) result in improved survival over single-agent gemcitabine. The median overall survival (OS) and progression-free survival (PFS) were 8.5 and 5.5 months in the gemcitabine/nab-paclitaxel group as compared to 6.7 and 3.7 months with single-agent gemcitabine group. Likewise, the median overall survival (OS) and progression-free survival (PFS) were 11.1 and 6.4 months in the FOLFIRINOX group as compared to 6.8 and 3.3 months with gemcitabine alone [2, 3]. However, the course of aggressive disease is unlikely to be altered by cytotoxic drugs alone, and addition of molecularly targeted agents is the focus of current investigations.

Previous studies showed that epidermal growth factor receptor (*EGFR*) aberrations are common in PCA and represent therapeutic targets [4]. Furthermore, the addition of erlotinib to gemcitabine resulted in a modest survival improvement over single-agent gemcitabine [5]. However, the association between clinical response to erlotinib and the presence of *EGFR* and *KRAS* mutations remains to be conclusively proven, and studies have yielded inconsistent results [6–9] However, tumors that respond to *EGFR* inhibitors may develop resistance, either due to mutant *KRAS*, development of secondary *EGFR* mutations, *c-met* amplification, or cross talk between *EGFR* and insulin-like growth factor-I receptor (IGF-1R) pathways [10].

Binding of IGF-1 to its receptor (IGF-1R) initiates downstream signals that activate PI3K/Akt/mTOR and MEK/Erk pathways, which stimulate cellular proliferation and induce drug resistance [11]. Inhibition of IGF-1R signaling enhanced the antitumor effect of gemcitabine and cisplatin in PCA xenografts and ovarian cancer cell lines, respectively [12, 13]. Furthermore, the addition of h7C10, anti-IGF-1R monoclonal antibody (mAB), to cetuximab, *EGFR* mAB, in A549 non-small cell lung cancer (NSCLC) xenograft models of wild-type *EGFR* and activated *RAS* mutation led to growth inhibition, unlike cetuximab alone [14].

MK-0646, humanized IGF-1 mAB, binds to IGF-1R. This binding inhibits IGF-1R autophosphorylation and downstream signaling activation of PI3K/Akt/mTOR and MEK/Erk pathways, leading to inhibition of cellular proliferation [15]. Our study was planned before the clinical trials of gemcitabine/nab-paclitaxel or FOLFIRINOX. Our purpose was to determine safety, tolerability, and outcomes of MK-0646 with gemcitabine ± erlotinib in advanced PCA.

## Methods

### Study design

This study was an open-label single-institution three-part clinical trial comprising a phase I dose escalation cohort, a phase II randomization cohort, and a phase II expansion cohort.

**Fig. 1:**
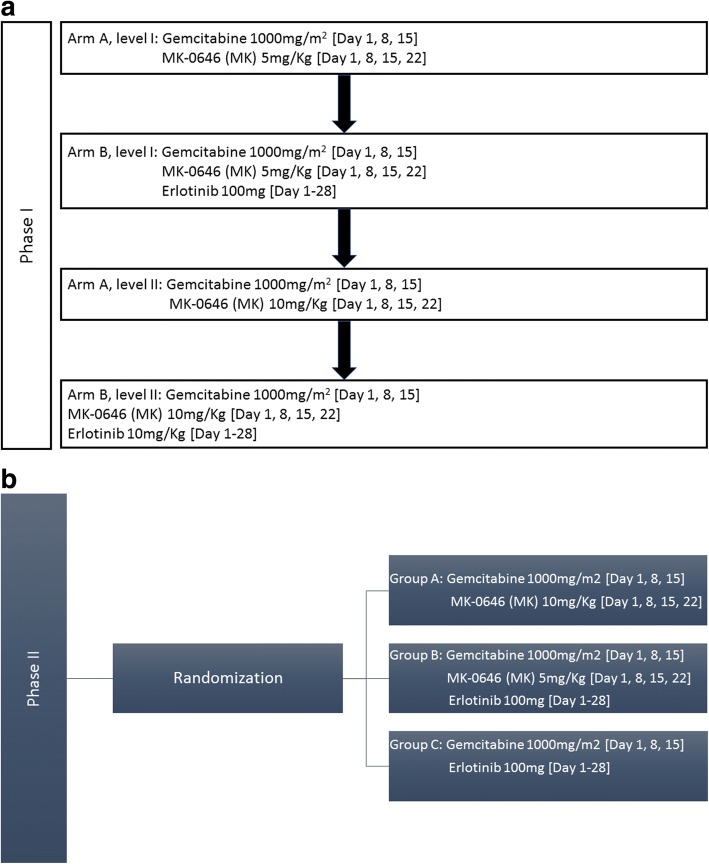
Study design schemes. **a** Phase I dose escalation trial scheme. **b** Phase II randomization trial scheme

In phase I, a 3 + 3 dose escalation design was used to determine the MK-0646 maximum tolerable dose (MTD) in combination with G (gemcitabine) (arm A) or G + E (erlotinib) (arm B). Gemcitabine was administered at 1000 mg/m^2^ over 100 min on days 1, 8, and 15 of a 28-day cycle, while erlotinib was administered orally at 100 mg daily. MK-0646 was administered intravenously at two dose levels: 5 mg/kg (level I) or 10 mg/kg (level II) on days 1, 8, 15, and 22. The MTD (i.e., recommended phase II dose (RP2D)) was defined as the highest dose that induced a dose-limiting toxicity (DLT) in < 2 patients among at least six patients. Patient enrollment in this phase was sequential, not randomized (Fig. 1a).

In phase II, patients were randomized into three arms: A (G + MK), B (G + MK + E), and C (G + E), where the RP2D from phase I was used for arms A and B. The primary endpoint was progression-free survival (PFS). A Bayesian adaptive randomization design was used where the first 45 patients were equally randomized among the three arms. As the trial progressed and data accrued, the randomization was planned in favor of the treatment arm with better PFS results. If at any point, the posterior probability of a given arm being better than other two arms was less than 10%, that arm was suspended. A minimum of 45 and a maximum of 78 patients were planned for enrollment.

In the expansion phase, additional patients were enrolled to receive G + MK for correlative studies. Plasma and tissue levels of IGF-1 were measured for phase II patients to assess the correlation between IGF-1 expression and OS. This clinical trial (NCT00769483) was approved by the Institutional Review Board, and all study participants signed an informed consent.

### Patient selection

The eligibility criteria included treatment naïve metastatic PCA; age > 18 years; Eastern Cooperative Oncology Group (ECOG) ≤ 1; adequate organ function; had measurable disease as defined by the Response Evaluation Criteria in Solid Tumor (RECIST) version 1.1; ≥ 6 months elapsed since completion of previous therapy; and patients who enrolled in phase II cohorts were required to had biopsies for correlative studies.

We excluded patients who had prior systemic therapy, had brain metastases, were pregnant or nursing, had uncontrolled illness that would limit study compliance, and had another cancer, except treated basal or squamous cell skin carcinoma or cervical carcinoma in situ, or if patient had been disease-free for > 2 years.

### Safety and efficacy assessments

All patients underwent a complete medical evaluation, with assessment of ECOG status, adverse events, and hematological and organ function laboratory analysis on a weekly basis during the first 2 cycles and once every cycle thereafter unless the patient experienced treatment-emergent adverse events (TEAE). Radiological assessment of tumor response according to RECIST version 1.1 criteria was performed every 2 cycles.

### Dose-limiting toxicity

All toxic effects were graded according to the National Cancer Institute’s Common Terminology Criteria for Adverse Events v3.0. Each patient was evaluated for DLT after the first cycle; DLT was defined as grade 4 (G4) neutropenia for ≥ 7 days, febrile neutropenia (≥ G3 neutropenia of any duration with fever ≥ 38.5 °C), G4 thrombocytopenia, or ≥ G3 non-hematological toxicity excluding hyperglycemia, skin rash, nausea, vomiting, or diarrhea, unless these occurred despite maximal prophylaxis or treatment. For all G3/4 TEAE, treatment was withheld until the patient’s symptoms resolved or returned to G1. Any dose interruption for > 14 days because of TEAE was considered DLT.

### Plasma and tissue IGF-1 assay

Blood samples were collected, anticoagulated, and centrifuged; then, IGF-1 was measured in plasma by Quantikine Human IGF-1 enzyme-linked immunosorbent assay Kit. The expression of IGF-1 in tissue was assessed by using reverse transcription reaction. Pre-amplification techniques were used to amplify targeted cDNA prior to quantitative polymerase chain reaction analysis. The generated data were then analyzed using LightCycler^®^ 480 software.

### Statistical analysis

Patient characteristics were summarized using median (range) for continuous variables and frequency (percentage) for categorical variables. The probabilities of overall survival (OS) and progression-free survival (PFS) were estimated using the Kaplan-Meier method. OS was defined as the time interval between start of treatment and death date. Patients who were alive were censored at the last follow-up date. PFS was defined as the time interval between start of treatment and date of disease progression or death. Patients who were alive and without disease progression were censored at the last follow-up date. Log-rank tests were used to assess the differences in OS and PFS between treatment arms. All statistical analyses were conducted by using SAS version 9.2 (SAS Institute Inc.) and S plus software version 8 (TIBCO Software, Inc). *P* value < 0.05 was considered significant.

## Results

### Study participants

A total of 81 patients were enrolled over the three cohorts between December 2008 and October 2013. In phase I, 22 patients were enrolled but only 21 were treated, as one patient withdrew consent and was never treated with the study drug. For phase II randomization cohort, 50 patients were enrolled and 45 patients were evaluable for evaluation. Of the 5 non-evaluable patients, 1 patient did not pass the screen and was never treated, 1 patient withdrew consent after being randomized to G + E, and 3 patients were not randomized or treated. The remaining 45 patients were equally randomized among the three arms. An additional 9 patients were enrolled in the expansion cohort and were treated with G + MK. Overall, 75 patients were treated in phase I and II. Patients’ demographics and clinical characteristics are summarized in Table 1.

**Table 1:** Patients demographics and clinico-pathological characteristics for phase I and II enrolled pancreatic cancer patients (*N* = 75)

	Enrolled patients (*N* = 75) (%)
Age group	
Median (range)	62.8 (44–83)
< 60	27 (36%)
≥ 60	48 (64%)
Sex	
Male to female ratio	1.7:1
Female	28 (37.3%)
Male	47 (62.7%)
Race	
White	61 (81.3%)
Black	4 (5.3%)
Hispanic	4 (5.3%)
Asian	4 (5.3%)
Others	2 (2.7%)
Tumor differentiation	
Moderate	22 (29.3%)
Poor	23 (30.7%)
Unknown	30 (40%)
ECOG	
0	8 (10.7%)
1	67 (89.3%)
Tumor location	
Head	22 (29.3%)
Body	32 (42.7%)
Tail	19 (25.3%)
Unknown	2 (2.7%)
Site of metastasis	
Liver	61 (75.3%)
Lung	10 (12.3%)
Peritoneal	15 (18.5%)
Others	4 (5.3%)
CA19-9 (U/ml)	
Median (range)	58,159.1 (0.9–58,160)
≤ 35	12 (16%)
> 35	63 (84%)
Previous treatment	
De novo	69 (92%)
Surgery	4 (4.9%)
Chemotherapy	4 (4.9%)

### Phase I dose escalation

The first 3 patients were treated with G + MK 5 mg/kg (arm A, level I) and completed the first cycle without DLT. The next 3 patients were treated with the same regimen plus erlotinib (arm B, level I) without DLT. The next 3 patients were treated with G + MK 10 mg/kg (arm A, level II) without DLT. However, when erlotinib was added to this regimen in the next 3 patients, 1/3 developed DLT in the form of G3 febrile neutropenia (arm B, level II). An additional 3 patients were enrolled in arm A, level II, and another 3 in arm B, level II. While none of these 3 additional patients in arm A, level II, developed DLT, 1/3 patients enrolled in arm B, level II, developed DLT that required treatment interruption for > 14 days. Therefore, an additional 3 patients were enrolled under arm B, level I, without DLT. Thus, MK-0646 10 mg/kg was declared to be the MTD in combination with gemcitabine and 5 mg/kg the MTD in combination with G + E (Fig. 1b).

### Phase II randomization and expansion cohort

**Fig. 2:**
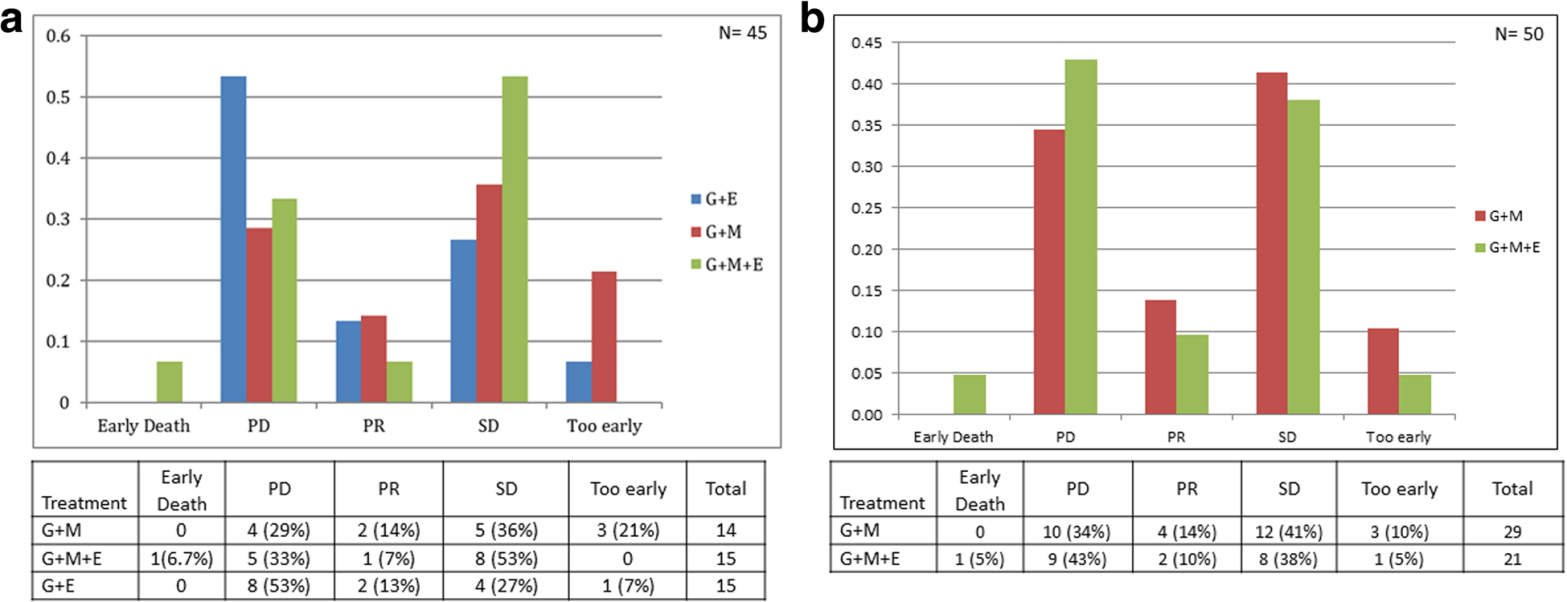
Treatment response within the treatment arms. **a** Treatment response rates between patients treated with gemcitabine + MK-0646, gemcitabine + MK-0646 + erlotinib, and gemcitabine + erlotinib alone as a part of phase II randomization cohort. **b** Treatment response rates for all patients treated with gemcitabine + MK-0646 (10 mg/kg) compared with gemcitabine + MK-0646 (5 mg/kg) + erlotinib as a part of phase I and II (randomization and expansion) cohorts. G, gemcitabine; M, MK-0646; E, erlotinib; PD, progressive disease; PR, partial response; SD, stable disease

The median number of cycles administered was 2 (range 1–11) in arm A, 2 (range 1–23) in arm B, and 2 (range 1–7) in arm C. Treatment response was evaluated for all phase II patients except for 1 patient who was treated with G + MK. The responses are summarized in Fig. 2a.

**Fig. 3:**
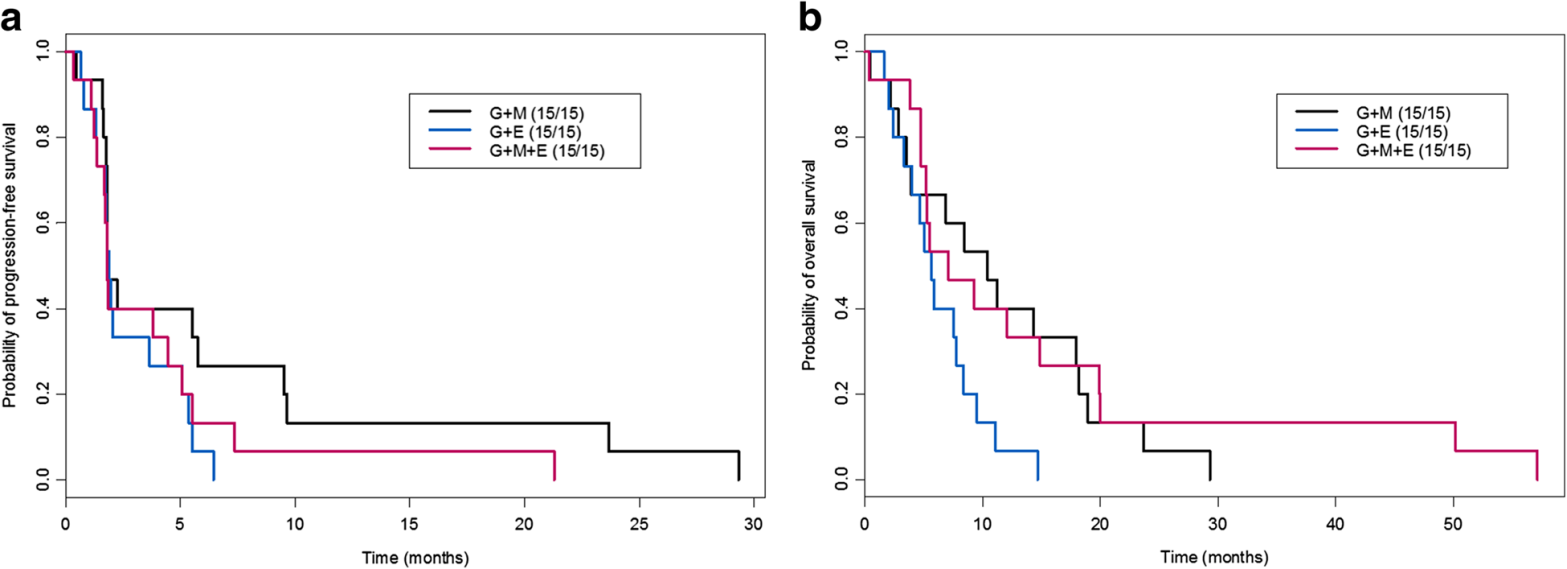
Kaplan-Meier curves for the three arms of the phase II randomization cohort. **a** Kaplan-Meier progression-free survival curves between the three arms of the phase II randomization cohort. **b** Kaplan-Meier overall survival curves between the three arms of the phase II randomization cohort. G, gemcitabine; M, MK-0646; E, erlotinib

Among the 45 randomized patients, the estimated median PFS was 1.8 months (95% confidence interval [CI] 1.8–9.7) for arm A, 1.8 months (95% CI 1.7–5.5) for arm B, and 1.9 months (95% CI 1.8–5.4) for arm C. The difference between arms A and C was marginally significant (*P* = 0.09), but the difference between arms A and B was not significant (*P* = 0.20; Fig. 3a). Furthermore, the median OS was 10.4 months (95% CI 3.9–18.9) for arm A, 7.1 months (95% CI 5.2–20.0) for arm B, and 5.7 months (95% CI 4.0–9.5) for arm C. However, patients treated with G + MK had a significantly longer OS than patients treated with G + E (*P* = 0.02). Addition of erlotinib to G + MK did not improve OS (*P* = 0.60; Fig. 3b). We also computed the posterior probability of each arm in terms of PFS, and the probability of G + E arm was below the pre-defined threshold of 0.10. We therefore withheld patient enrollment in G + E arm and expanded enrollment to additional 9 patients for G + MK arm.

**Fig. 4:**
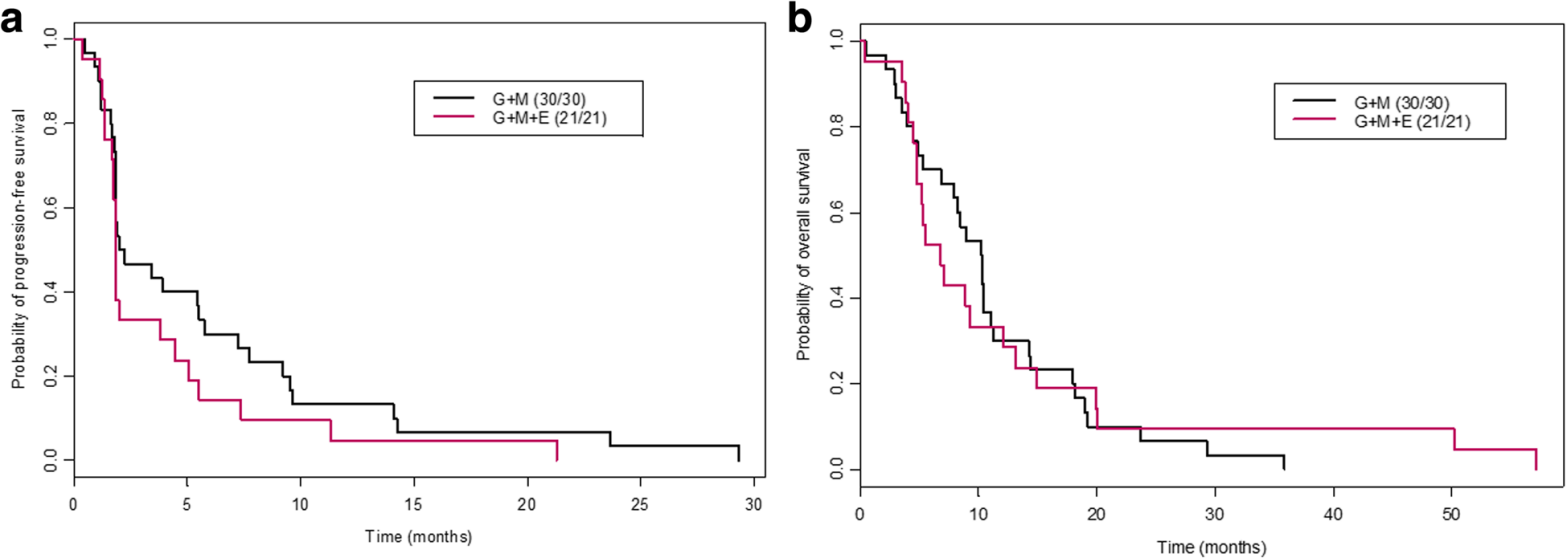
Kaplan-Meier curves for all patients treated with gemcitabine + MK-0646 compared with gemcitabine + MK-0646 + erlotinib during phase I and II (randomization and expansion) cohorts. **a** Kaplan-Meier progression-free survival curves for patients treated with gemcitabine + MK-0646 compared with gemcitabine + MK-0646 + erlotinib. **b** Kaplan-Meier overall survival curves for patients treated with gemcitabine + MK-0646 compared with gemcitabine + MK-0646 + erlotinib

During phase I and phase II combined, a total of 30 patients (6 from phase I and 24 from phase II) were treated with G + MK 10 mg/kg and 21 patients (6 from phase I and 15 from phase II) with G + MK + E 5 mg/kg. The median number of administered cycles was 2 (range 1–12) for arm A and 2 (range 1–23) for arm B. Responses to treatment for these patients are summarized in Fig. 2b. The estimated median PFS for phase I and phase II patients combined was 2.1 months (95% CI 1.8–7.2) for arm A and 1.8 months (95% CI 1.8–5.1) for arm B. The median OS was 10.3 months (95% CI 8.0–14.3) for arm A and 6.8 months (95% CI 4.8–14.9) for arm B (Fig. 4a, b).

In terms of subsequent therapies administered after first-line G + M or G + E, Additional file 1 shows that 53.3% of patients progressed on G + E received supportive care alone as compared with 40% of G + MK group.

### Treatment toxicity and tolerability

Additional file 2 summarizes the reported G3/4 TEAE in phase II cohorts. The most frequently reported grade 3 toxicity in group A were hyperglycemia (33.3%), thrombocytopenia (29.2%), leukopenia (20.8%), lymphopenia (20.8%), neutropenia (16.7%), and elevated AST (12.5%); in group B, toxicities noted were thrombocytopenia (53.3%), neutropenia (53.3%), leukopenia (33.3%), fatigue (26.7%), elevated ALT (20%), hyponatremia (20%), and acne-like rash (13.3%); and in group C, they were neutropenia (33.3%), leukopenia (33.3%), anemia (13.3%), and fatigue (13.3%). Furthermore, 40% of patients treated in group B developed G4 neutropenia, compared to 25% in arm A. Generally, 3 patients in arm A and 4 patients in arm B developed a DLT, in the form of G4 thrombocytopenia and G4 neutropenia for ≥ 7 days. None of the patients developed febrile neutropenia.

### IGF-1 as a predictive marker for MK-0646

Among phase II cohorts, 21 patients had available plasma and 23 patients had available tissue to measure the level of IGF-1. Although the mean level of plasma IGF-1 was higher in patients with OS ≥ 12 months, there was no significant correlation between plasma IGF-1 level and OS (*P* = 0.64). The same result was noted for the IGF-1 tissue expression (*P* = 0.87). However, the lack of significant effects could be due to the small sample of patients with OS ≥ 12 months (Additional file 3).

## Discussion

Based on our phase I dose escalation results, we determined that MK-0646 RP2D is 10 mg/kg in combination with gemcitabine and 5 mg/kg in combination with G + E. Notably, G + MK was associated with acceptable toxicity and longer OS than G + E in the phase II. Furthermore, addition of erlotinib to G + MK did not improve OS and PFS. The observed adverse events associated with MK-0646 were generally tolerable, the most frequently reported being hyperglycemia and hematological toxicities.

The IGF pathway is regulated by two ligands (IGF-1 and IGF-1I), two transmembrane receptors (IGF-1R and IGF-1IR), and up to ten IGF-binding proteins (IGFBPs). Binding of IGF-1 and IGF-1I to their receptors results in auto-activation of tyrosine kinases and autophosphorylation of tyrosines, including tyrosine 950 in the juxta-membrane, which can serve as the docking site for the insulin receptor substrates (IRS) and SHC. IRS-I stimulates the PI3K/AKT/mTOR, Src, and SHC pathways. Activation of the SHC pathway induces formation of the Grb-2/son of sevenless complex, which activates the p21 Ras and Raf/MEK/Erk pathways, leading to cellular proliferation [16].

We demonstrated that IRS-specific small interfering RNA inhibited activation of PI3K/AKT/mTOR in transfected PCA cells [17]. Therefore, investigating the IGF pathway in cancer is crucial. MK-0646, a humanized IGF-1 mAB, has been previously tested in several cancers [18–23]. To our knowledge, this is the first clinical trial to evaluate MK-0646 efficacy and safety in PCA patients.

The efficacy of IGF-1R inhibitors in the clinical setting is unproven. Although a few advanced solid tumors have responded to IGF-1R inhibitors, the majority showed no evidence of improvement. In a trial of the IGF-1R mAB [IMC-A12] ± cetuximab in metastatic colorectal carcinomas (MCRC), 1/41 patients had a partial tumor response (PR) with combined therapy, while no antitumor activity was observed in the monotherapy arm [24]. MK-0646 was tolerable in combination with cisplatin and etoposide in small cell lung cancer, but the clinical response was not meaningfully different from cisplatin and etoposide alone [23]. In contrast, 10/28 sarcoma patients treated with figitumumab, another IGF-1R mAB, had stable disease (SD) or PR [25]. In our study, addition of MK-0646 to G ± E yielded 53 and 36% SD rates, respectively, compared to 27% in patients treated with G + E.

Preclinical studies showed a promising effect of MK-0646 + cetuximab [14]. In a randomized phase II/III study evaluating the response to MK-0646 + cetuximab + irinotecan in MCRC, addition of MK-0646 did not improve OS or tumor response [21]. The same treatment combination in a phase I clinical trial in Japanese MCRC patients showed that the triple combination is well tolerated [19]. A randomized phase II/III study was initiated in the same population to evaluate safety, tolerability, and effectiveness of this triple combination, but the trial was terminated at the first interim analysis because the triple combination yielded significantly shorter OS and PFS [26]. Similarly, addition of cixutumumab, humanized IGF-1R mAB, to G + E did not improve PFS or OS compared with G + E alone in PCA [27]. These findings support our data wherein the addition of erlotinib did not result in any improvement in OS or PFS as compared with G + MK. In contrast, the combination of ganitumab, humanized IGF-1R mAB, with gemcitabine was tolerable and associated with a trend in OS improvement compared to gemcitabine alone in PCA [28]. This was also noted in our study with G + MK. Although we have demonstrated that G + M demonstrated an improved OS as compared with G + E, the PFS was not different between these arms. One possible explanation for this discordance is that a greater number of patients with G + M received second-line therapy as compared with G + E. More patients in the G + E arm transitioned to supportive care or hospice due to deterioration in performance status.

These findings highlight the need to identify IGF-1R mAB response predictive biomarkers. A significant correlation between circulating level of free IGF-1 and response to figitumumab was reported in patients with NSCLC [29]. Furthermore, high tissue expression of IGF-1 was a surrogate biomarker for response to MK-0646 [26]. Atzori et al. evaluated the safety and tolerability of MK-0646 in patients with advanced solid tumors expressing IGF-1R and found that, although MK-0646 was well tolerated, most tumors did not respond to treatment despite their IGF-1R expression [22]. In our study, there was no significant correlation between IGF-1 level and OS rate; however, this could be due to the limited size.

Our study has several limitations. First, it is a single institutional experience with a limited sample size in each treatment arm. Also, in our study we compared MK-0646 with gemcitabine ± erlotinib. Although, gemcitabine alone is currently used for advanced pancreatic cancer patients with ECOG PS of 2, at the current time, first-line regimens of choice for patients with good performance status include FOLFIRINOX and gemcitabine/nab-paclitaxel. Despite these limitations, we hypothesize that the combination of MK-0646 with the above combination regimens may result in favorable outcomes, given its low toxicity and overall impact on survival resulting from sequential therapies. Multicenter trials exploring MK-0646 with combination chemotherapy regimens are warranted.

## Conclusion

Although addition of MK-0646 to gemcitabine resulted in an OS improvement and tolerable toxicities as compared with gemcitabine plus erlotinib, a greater fraction of patients receiving gemcitabine + MK-0646 received second-line therapies as compared with gemcitabine and erlotinib. Future clinical trials are necessary to identify the impact of MK-0646 addition to gemcitabine/nab-paclitaxel and FOLFIRINOX.

## Additional files

Additional file 1: Table summary of various post-study treatment approaches. (DOCX 18 kb)

Additional file 2: The most commonly reported grade 3 and 4 toxicity in patients treated with gemcitabine + MK-0646 versus gemcitabine + erlotinib with and without MK-0646. (DOCX 21 kb)

Additional file 3: A box plot flow to identify variations in insulin like growth factor 1 expression in plasma and tissue between patients with overall survival rate ≥ 12 months versus those with short survival defined as < 12 month. (TIF 107 kb)

## Abbreviations

DLT: Dose-limiting toxicity
E: Erlotinib
ECOG: Eastern Cooperative Oncology Group
EGFR: Epidermal growth factor receptor
G: Gemcitabine
IGF-1R: Insulin-like growth factor-1 receptor
IGFBPs: Insulin-like growth factor binding proteins
IRS: Insulin receptor substrates
mAB: Monoclonal antibody
MCRC: Metastatic colorectal carcinomas
MTD: Maximum tolerable dose
NSCLC: Non-small cell lung cancer
OS: Overall survival
PCA: Pancreatic cancer
PFS: Progression-free survival
PR: Partial response
RECIST: Response Evaluation Criteria in Solid Tumor
RP2D: Recommended phase II dose
SD: Stable disease
TEAE: Treatment-emergent adverse events
