# Supplementary material for: Randomized, phase I/II study of gemcitabine plus IGF-1R antagonist (MK-0646) versus gemcitabine plus erlotinib with and without MK-0646 for advanced pancreatic adenocarcinoma
Source: J Hematol Oncol. 2018 May 30;11:71. doi: 10.1186/s13045-018-0616-2 (PMC5975422; doi:10.1186/s13045-018-0616-2)
Supplement: Supplementary file 1 — Table summary of various post-study treatment approaches. (DOCX 18 kb) [file 13045_2018_616_MOESM1_ESM.docx]

**Supplemental table 1. Various post-study treatment approaches according to the treatment group**

|  | **(Gemcitabine + MK-0646)**  **(N=30) (%)** | **(Gemcitabine + Erlotinib)**  **(N=15) (%)** |
| --- | --- | --- |
| Supportive care only | 12 (40%) | 8 (53.3%) |
| Gemcitabine + Cisplatin | 2 (6.7%) | ------- |
| Gemcitabine + Erlotinib | 1 (3.3%) | 1 (6.7%) |
| Gemcitabine + Cisplatin + maintenance Capecitabine | 1 (3.3%) | ------- |
| Gemcitabine + Capecitabine + Taxotere | 1 (3.3%) | ------- |
| Oxaliplatin + Capecitabine (XELOX) | 1 (3.3%) | ------ |
| Leucovorin + 5FU + Oxaliplatin (FOLFOX) | 6 (20%) | 2 (13.3%) |
| Gemcitabine + Oxaliplatin + Capecitabine | 1 (3.3%) | ------ |
| Radiotherapy concurrent with Capecitabine | 2 (6.7%) | 1 (6.7%) |
| 5-fluorouracil + Leucovorin + Irinotecan + Oxaliplatin (FOLFIRINOX) | 1 (3.3%) | 2 (13.3%) |
| Gemcitabine + Erlotinib + MK-0646 | ------- | 1 (6.7%) |
| Gemcitabine + abraxane | 2 (6.7%) | ------- |
