# Supplementary material for: Randomized, phase I/II study of gemcitabine plus IGF-1R antagonist (MK-0646) versus gemcitabine plus erlotinib with and without MK-0646 for advanced pancreatic adenocarcinoma
Source: J Hematol Oncol. 2018 May 30;11:71. doi: 10.1186/s13045-018-0616-2 (PMC5975422; doi:10.1186/s13045-018-0616-2)
Supplement: Supplementary file 2 — The most commonly reported grade 3 and 4 toxicity in patients treated with gemcitabine + MK-0646 versus gemcitabine + erlotinib with and without MK-0646. (DOCX 21 kb) [file 13045_2018_616_MOESM2_ESM.docx]

**Supplemental table 2. Grade 3 and 4 treatment related adverse events under each Phase II treatment arm.**

| Adverse events | Group A  (Gemcitabine + MK-0646)  (N=24) (%) | | Group B  (Gemcitabine + MK-0646 + Erlotinib)  (N=15) (%) | | Group C  (Gemcitabine + Erlotinib)  (N=15) (%) | |
| --- | --- | --- | --- | --- | --- | --- |
|  | **G 3** | **G4** | **G3** | **G4** | **G3** | **G4** |
| Hematological |  |  |  |  |  |  |
| Anemia | 2 (8.3%) | ------ | 1 (6.7%) | ------ | 2 (13.3%) | ------ |
| Leukopenia | 5 (20.8%) | ------ | 5 (33.3%) | ------ | 5 (33.3%) | 2 (13.3%) |
| Neutropenia | 4 (16.7%) | 6 (25%) | 8 (53.3%) | 6 (40%) | 5 (33.3%) | ------ |
| Thrombocytopenia | 7 (29.2%) | 1 (4.2%) | 8 (53.3%) | 1 (6.7%) | 1 (6.7%) | 1 (6.7%) |
| Lymphopenia | 5 (20.8%) | ------ | 1 (6.7%) | ------ | 1 (6.7%) | ------ |
|  |  |  |  |  |  |  |
| Non-hematological |  |  |  |  |  |  |
| Fatigue | 2 (8.3%) | ------ | 4 (26.7%) | ------ | 2 (13.3%) | ------ |
| Elevated ALK | 1 (4.2%) | ------ |  | ------ | ------ | ------ |
| Elevated ALT | 2 (8.3%) | ------ | 3 (20%) | ------ | ------ | ------ |
| Elevated AST | 3 (12.5%) | ------ | 1 (6.7%) | ------ | ------ | ------ |
| Elevated INR |  |  | 1 (6.7%) |  |  |  |
| Hyperkalemia | 1 (4.2%) | ------ | ------ | ------ | ------ | ------ |
| Hyponatremia | 1 (4.2%) | ------ | 3 (20%) |  | ------ | ------ |
| Atrial fibrillation | 1 (4.2%) | ------ | ------ | ------ | ------ | ------ |
| Cardiac general | 1 (4.2%) |  | ------ | ------ | ------ | ------ |
| Hyperglycemia | 8 (33.3%) | ------ | 1 (6.7%) | ------ | ------ | ------ |
| Anorexia | 1 (4.2%) | ------ | ------ | ------ | 1 (6.7%) | ------ |
| Nausea | 1 (4.2%) | ------ | 1 (6.7%) | ------ | 1 (6.7%) | ------ |
| GIT Hemorrhage | 1 (4.2%) | ------ | ------ | ------ | ------ | ------ |
| Diarrhea | ------ | ------ | 1 (6.7%) | ------ | ------ | ------ |
| Thrombosis | ------ | ------ | 1 (6.7%) | ------ | ------ | ------ |
| Pulmonary embolism | ------ | ------ | 1 (6.7%) | 1 (6.7%) | ------ | ------ |
| Acne-like rash | ------ | ------ | 2 (13.3%) | ------ | ------ | ------ |
| Infection | ------ | ------ | 1 (6.7%) | ------ | ------ | ------ |
