# Supplementary figures and images for: Randomized, phase I/II study of gemcitabine plus IGF-1R antagonist (MK-0646) versus gemcitabine plus erlotinib with and without MK-0646 for advanced pancreatic adenocarcinoma
Source: J Hematol Oncol. 2018 May 30;11:71. doi: 10.1186/s13045-018-0616-2 (PMC5975422; doi:10.1186/s13045-018-0616-2)

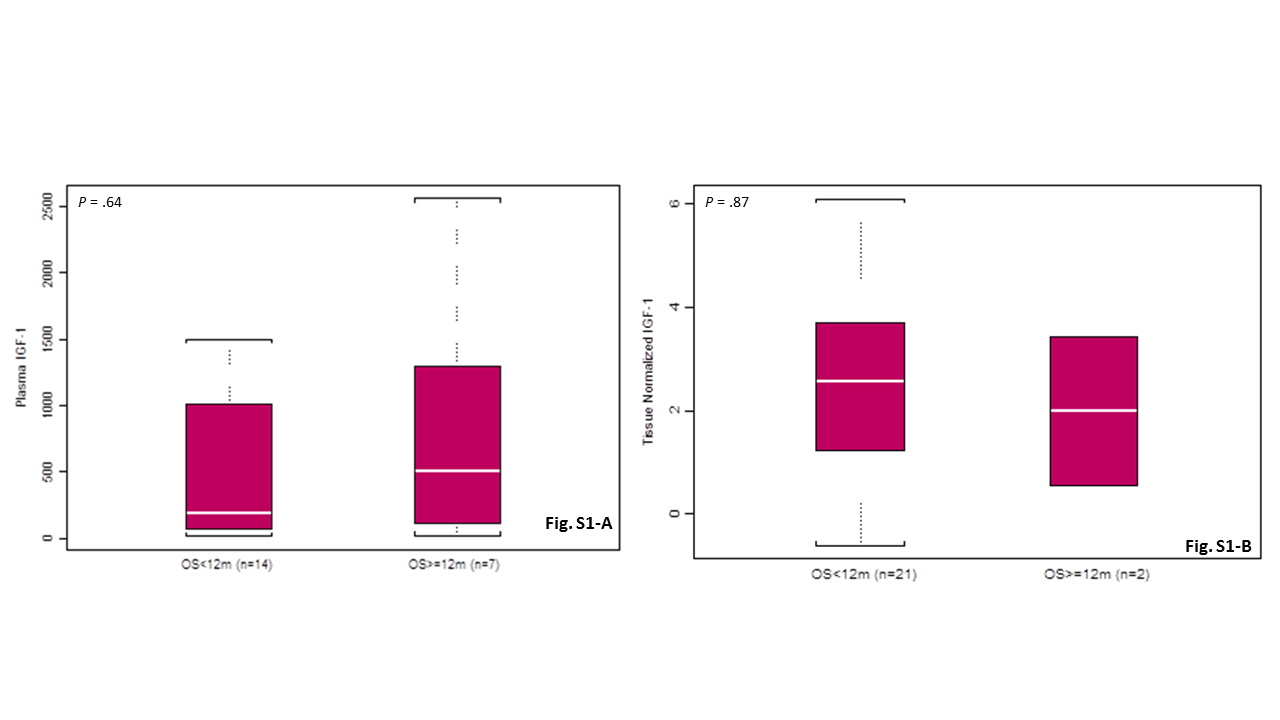

Supplement: Supplementary file 3 — A box plot flow to identify variations in insulin like growth factor 1 expression in plasma and tissue between patients with overall survival rate ≥ 12 months versus those with short survival defined as < 12 month. (TIF 107 kb) [file 13045_2018_616_MOESM3_ESM.tif]
